# Ballistic analysis of high-performance armor steel by numerical simulation

**DOI:** 10.1038/s41598-024-62482-5

**Published:** 2024-05-20

**Authors:** Deda Li, Feng Huang, Binzhi Ren, Wei Zhang, Junjie Xiong, Binjun Zhou, Xun Guo

**Affiliations:** 1 School of Mechanical and Vehicle Engineering, West Anhui University, Luan, 237012 China; 2 Hubei Key Laboratory of Advanced Technology for Automotive Components, Wuhan University of Technology, Wuhan, 430070 China; 3 School of Metallurgy and Materials Engineering, Chongqing University of Science and Technology, Chongqing, 401331 China

**Keywords:** Ballistic performance, Numerical simulation, Mechanical property effect, Predictive model, Materials science, Mathematics and computing

## Abstract

In order to establish a connection between the ballistic performance and mechanical properties of armor steel, a ballistic simulation model was developed and subsequently validated for accuracy and reliability. The mechanical properties of the target plate were described using the Johnson–Cook constitutive relation. An analysis was conducted to investigate the impact of the J–C parameters of the target plate on its ballistic performance, revealing a strong linear relationship between them. Subsequently, a mathematical model represented as H = 14.82 − 0.0048A − 0.0023B + 5.95n − 81.3C was derived, and its accuracy was demonstrated to exceed 90%. This mathematical model can effectively predict the ballistic performance of the armor steel, even when its mechanical properties undergo variations during the production process. This prediction capability significantly contributes to reducing research costs and time.

## Introduction

To meet the protective requirements of armored equipment, armor steel needs to possess high strength, high hardness, and good toughness^1^. When developing and applying new high-performance armor steel, the relationship between its mechanical properties and ballistic performance becomes a central focus.

Hardness plays a significant role in determining the failure model of the target when subjected to impacts. Woodward^2^ conducted a study on the relationship between hardness and the penetration behavior of armor steel against armor-piercing projectiles. The research revealed that as hardness increases, the ballistic response of the target can be categorized into four stages, each characterized by a distinct failure type. Investigating armor steels with hardness ranging from 300 to 600 HB against various projectile types, Ryan^3^ observed that the projectile type exerts a notable influence on the relationship between hardness and penetration resistance.

Regarding strength, researchers propose that higher strength leads to better resistance against small projectiles^4–6^. Børvik^7^, in an investigation of five different armor steels with varying yield strengths, determined a significant correlation between penetration resistance and yield strength by examining the limit ballistic velocity. However, other studies^8–11^ indicate that the aforementioned relationship between strength and ballistic performance is not universally applicable, particularly when the target is impacted by different projectile types. Apart from hardness and strength, the ductility of armor steel also affects its ballistic performance by absorbing impact energy^12–14^.

Numerical simulation serves as an effective method for analyzing the penetration behavior of target plates^15^. Cao^16^ employed this method to study the penetration process and corresponding failure mechanisms of a composite armor against armor-piercing projectiles, verifying simulation accuracy and optimizing parameters such as the order and thickness of laminates. Scazzosi^17^ investigated the ballistic performance of a protective armor composed of high-strength steel and aluminum alloy using numerical simulation. The study discussed the influences of the strain rate hardening parameter *C* and the thermal softening parameter *m*, while also analyzing the penetration process and corresponding energy dissipation mechanisms.

As highlighted previously, researchers worldwide have extensively investigated the ballistic performances of various armor steels from multiple perspectives. Enhancements in composition optimization and structural innovation^18,19^ have significantly improved the mechanical properties of armor steel, while advancements in heat treatment^20^ have further bolstered its ballistic performance. However, during actual production, the mechanical properties of armor steel tend to fluctuate within a certain range, consequently affecting the variability in ballistic performance. Since conducting live ammunition tests requires specialized facilities and dedicated personnel, it becomes impractical and expensive to evaluate the ballistic properties of every batch of armor steel. Therefore, this study utilizes numerical simulation to comprehensively investigate the relationship between the mechanical properties of a specific 4.6 mm armor steel and its corresponding ballistic performance. Consequently, a predictive mathematical model is established, enabling the accurate prediction of the armor steel’s ballistic performance. This approach significantly reduces the research costs and time involved. Furthermore, the methodology employed in establishing the predictive mathematical model in this paper can serve as a guiding framework for researching other protective materials.

## Materials and methods

### Ballistic performance tests

**Figure 1:**
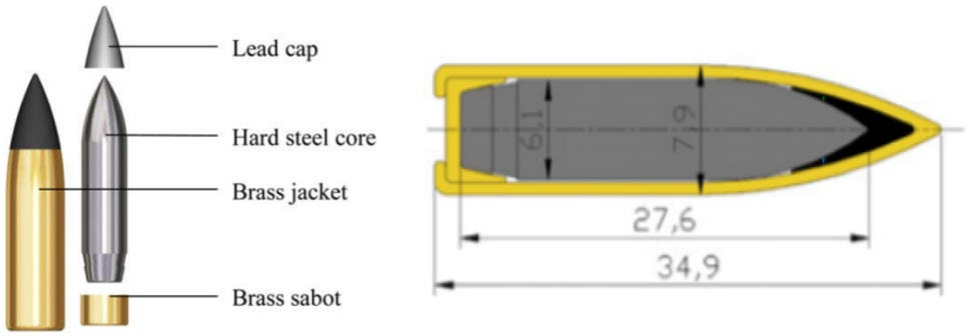
Schematic drawing, geometry, and cross-section picture of the 7.62 mm bullet (Units in mm)^7^.

All ballistic performance tests were conducted under room temperature conditions. The projectile used in this study was the standard 7.62 mm armor-piercing (AP) ammunition, as depicted in Fig. 1^7^. It is worth noting that the actual diameter of this bullet is approximately 7.9 mm, although it is commonly referred to as 7.62 mm. The dimensions of the target plate were 900 mm × 900 mm × 4.6 mm. Bullet velocity was measured using the GD-93 multi-purpose horizontal velocimeter throughout the tests. To ensure accuracy, three shooting tests were conducted on the target plate at each shooting distance.

**Figure 2:**
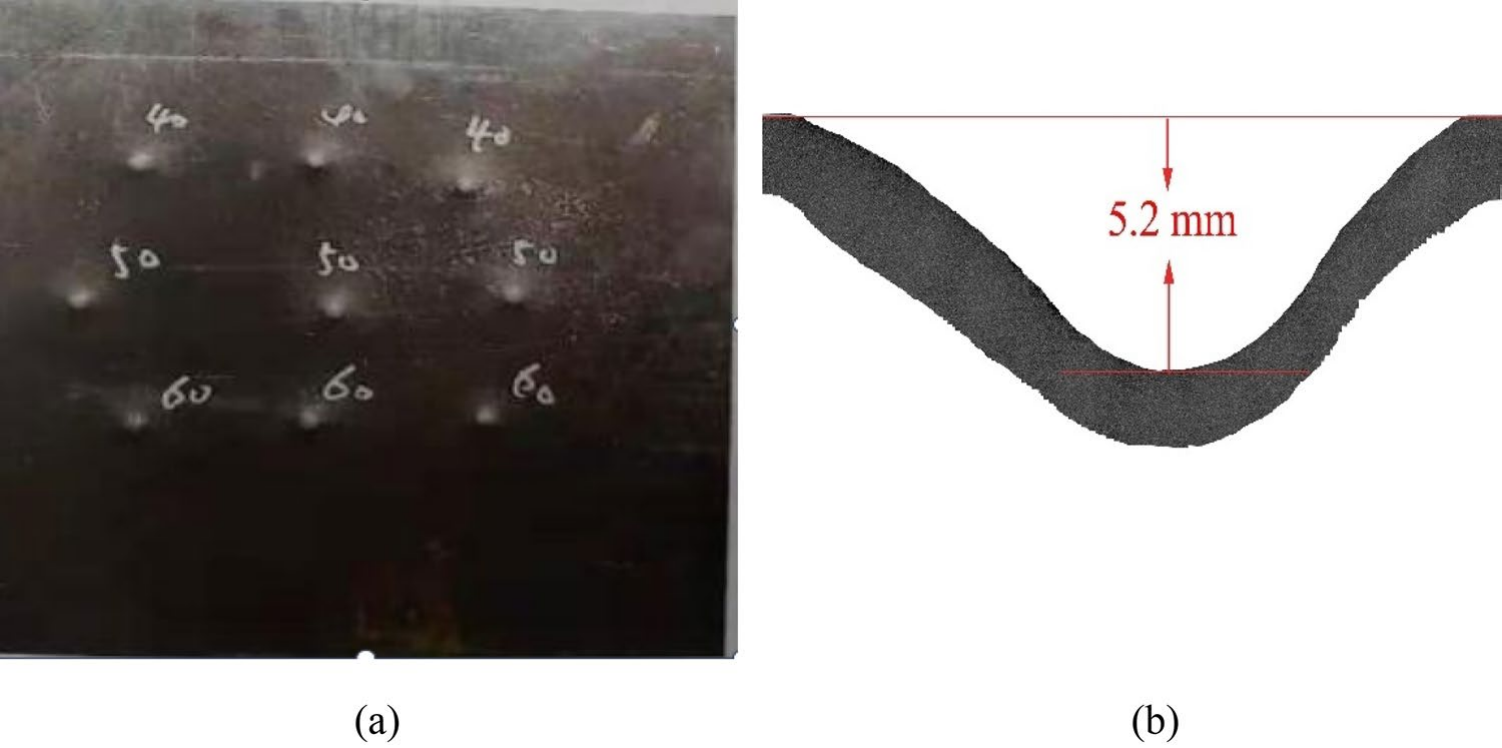
Appearance of the target plate after the shooting test: **a** the back, **b** the crater depth of Test-3.

Figure 2 illustrates the target plate’s appearance after the shooting tests, with the numbers 40, 50, and 60 corresponding to the respective shooting distances of 40 m, 50 m, and 60 m. It is observed that nine indentations were formed on the target plate, but none of them resulted in complete penetration. The crater depth of each pit was measured and recorded in Table 1. In the table, *v*_25_ represents the bullet velocity measured at a distance of 25 m from the shooting location. Based on prior experience, the bullet’s velocity decreases by approximately 10 m/s for every 10 m traveled in the air. Hence, the chosen impact velocity for the simulation (820 m/s) is nearly equivalent to the velocity observed when the shooting distance is 40 m. For the 40 m shooting distance, the average crater depth (H) of the impacted target plate, as calculated from Table 1, is 4.17 mm.

**Table 1:** Shooting results.

Specimen	Distance (m)	*v*_25_ (m/s)	Crater depth (mm)
Test-1	60	824	3.7
Test-2	60	829	3.8
Test-3	60	826	5.2
Test-4	50	833	4.1
Test-5	50	830	4.0
Test-6	50	829	4.4
Test-7	40	832	3.8
Test-8	40	832	4.6
Test-9	40	829	4.1

Tabulated data in Table 1 reveals that the crater depth generally decreases with increasing shooting distance, except for test-3, test-6, and test-7. The abnormal data observed in these three tests may be attributed to the following factors. Firstly, due to the non-uniform microstructure resulting from heat treatment, the mechanical and ballistic properties of the target plate vary across different locations. Secondly, residual stress is present in the plate and is not uniformly distributed, a phenomenon that has been shown to significantly impact ballistic performance^21^. Lastly, errors introduced during the shooting test may have contributed to the anomalous results.

### Computational material models

#### Constitutive relation

In this study, the Johnson–Cook constitutive relation (J–C), a well-established and reliable model as demonstrated in previous studies^22–24^, was selected. The equivalent stress (*σ*) is mathematically represented as follows:

1 $$ \sigma = \left( {A + B\varepsilon^{n} } \right)\left( {1 + C{\text{ln}}\dot{\varepsilon }^{ * } } \right)\left( {1 - \left( {T^{ * } } \right)^{m} } \right) $$

In the equation, *ε* represents the equivalent plastic strain, while *A*,* B*,* n*, *and C* denote the material constants^25^. The dimensionless plastic strain rate is described as $\left( {\dot{\varepsilon }^{ * } = \dot{\varepsilon }/\dot{\varepsilon }_{0} } \right)$, where $\dot{\varepsilon }_{0}$ is a user-defined reference strain rate. The homologous temperature is defined as T^*^ = (*T-T*_*r*_)/(*T*_*m*_*-T*_*r*_), where *T* represents the absolute temperature, *T*_*r*_ is the room temperature, and *T*_*m*_ is the melting temperature.

In Eq. (1), the J–C parameters are divided into two components: quasi-static and dynamic parameters. If we disregard the temperature effects, Eq. (1) can be simplified as follows:

2 $$ \sigma = (A + B\varepsilon^{n} )(1 + C{\text{ln}}\dot{\varepsilon }^{ * } ) $$

#### Material tests and identification of material constants

To determine the quasi-static parameters (*A*, *B*, and *n*) of the target plate, a quasi-static compression test at room temperature was conducted. A cylindrical specimen (*Φ*5 × 4 mm) was selected and cut from the target plate for this purpose. The corresponding stress (***σ***), strain (***ɛ***) and strain rate ($\dot{\varepsilon }$) can be calculated as

3 $$ \left\{ {\begin{array}{*{20}l} {\sigma = 4P/\pi d_{0}^{2} } \hfill \\ {\varepsilon = (l_{0} - l)/l_{0} } \hfill \\ {\dot{\varepsilon } = \Delta \varepsilon /\Delta t} \hfill \\ \end{array} } \right. $$

**Figure 3:**
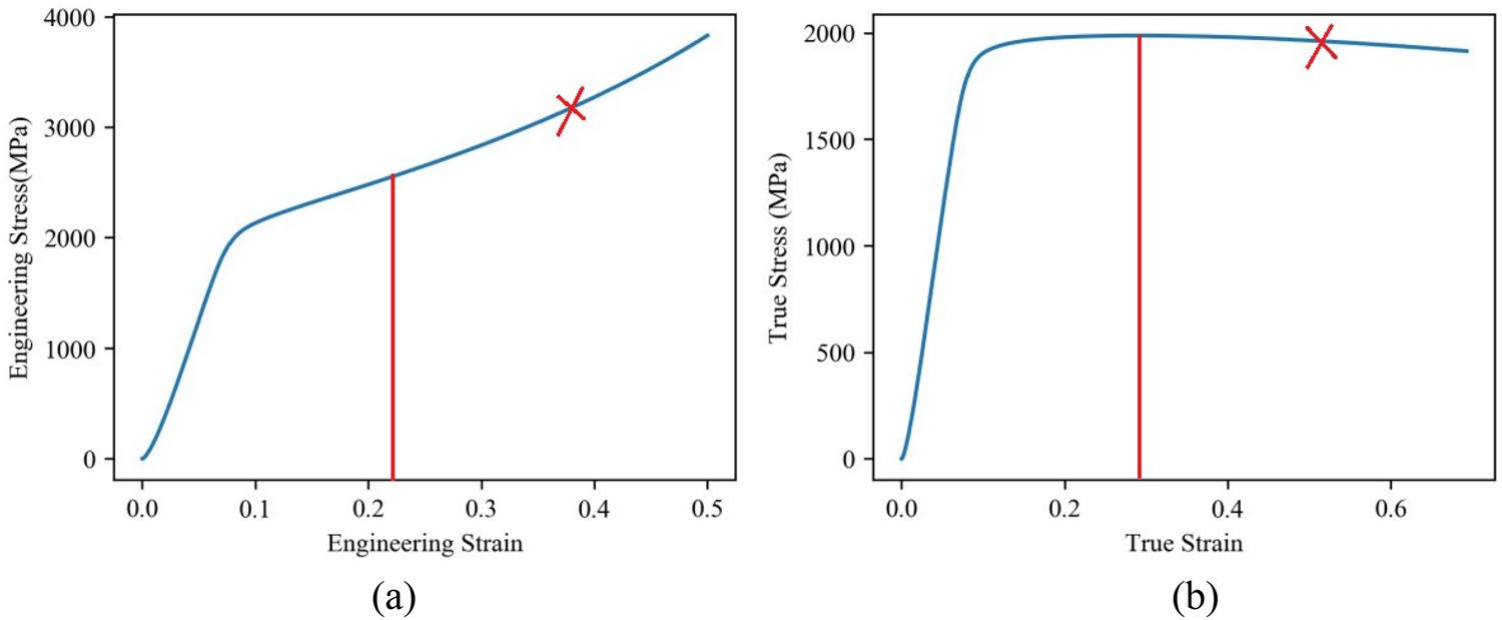
Stress–strain curves of the armor steel from quasi-static compression test: **a** Engineering Stress–strain curve, **b** True Stress–strain curve.

In the equation, *d*_0**,**_ *l*_0_, and *l* denote the initial diameter, initial length, and current length of the specimen, respectively, while *P* represents the applied pressure. Figure 3a illustrates the engineering stress–strain curve obtained from the test. At the later stage of the compression test, the specimen has a significant bulging phenomenon, and the subsequent stress–strain curve is invalid, that. However, due to the changes in specimen diameter and length during compression, the stress and strain directly measured by the equipment do not accurately reflect the true stress and strain generated in the specimen. Consequently, the true stress (s) and strain (e) should be calculated from the engineering stress (*σ*) and strain (*ε*) using Eq. (4). The corresponding true stress–strain curve, calculated from these equations, is depicted in Fig. 3b. As observed, the true stress is notably lower than the engineering stress, while the true strain is slightly higher than the engineering strain.

4 $$ \left\{ {\begin{array}{*{20}l} {s = \sigma (1 - \varepsilon )} \hfill \\ {e = - {\text{ln}}(1 - \varepsilon )} \hfill \\ \end{array} } \right. $$

**Figure 4:**
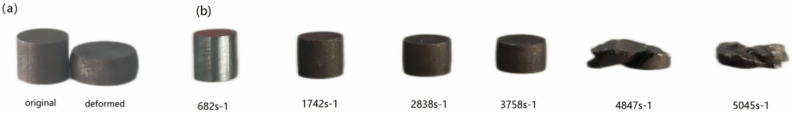
Comparison of the original and deformed specimens: **a** Quasi-static compression test, **b** SHPB tests.

Figure 4a displays the original and deformed specimen in the quasi-static compression test. The figure reveals that the specimen takes on a drum-like shape after compression due to significant friction between the specimen and equipment, which impedes transverse deformation at both ends. Furthermore, no cracks are observed on the specimen surface, even at a compression strain of 0.5, indicating the good ductility of the armor steel.

In quasi-static compression, the nominal strain rate is merely 1 × 10^−3^ s^−1^, rendering the strain rate effect negligible. Consequently, Eq. (2) can be simplified as Eq. (5) for the purpose of calculating the quasi-static parameters. Notably, parameter *A* corresponds to the yield stress. Additionally, Eq. (6) can be derived from Eq. (5). By utilizing the calculated results presented in Fig. 3b and fitting the corresponding data using Eq. (6), as shown in Fig. 6a, the values for the quasi-static parameters *B* and *n* can be determined, as listed in Table 2.

5 $$ \sigma = A + B\varepsilon^{n} $$

6 $$ {\text{ln}}(\sigma - A) = \ln B + n{\text{ln}}\varepsilon $$

**Table 2:** J–C parameters for the target plate and AP bullet^26^.

	*A* (MPa)	*B* (MPa)	*n*	*C*
Target plate	1630	1050	0.32	0.024
Hard core	1900	1100	0.3	0.005
Lead cap	24	300	1.0	0.1
Brass jacket and sabot	206	505	0.42	0.001

**Figure 5:**
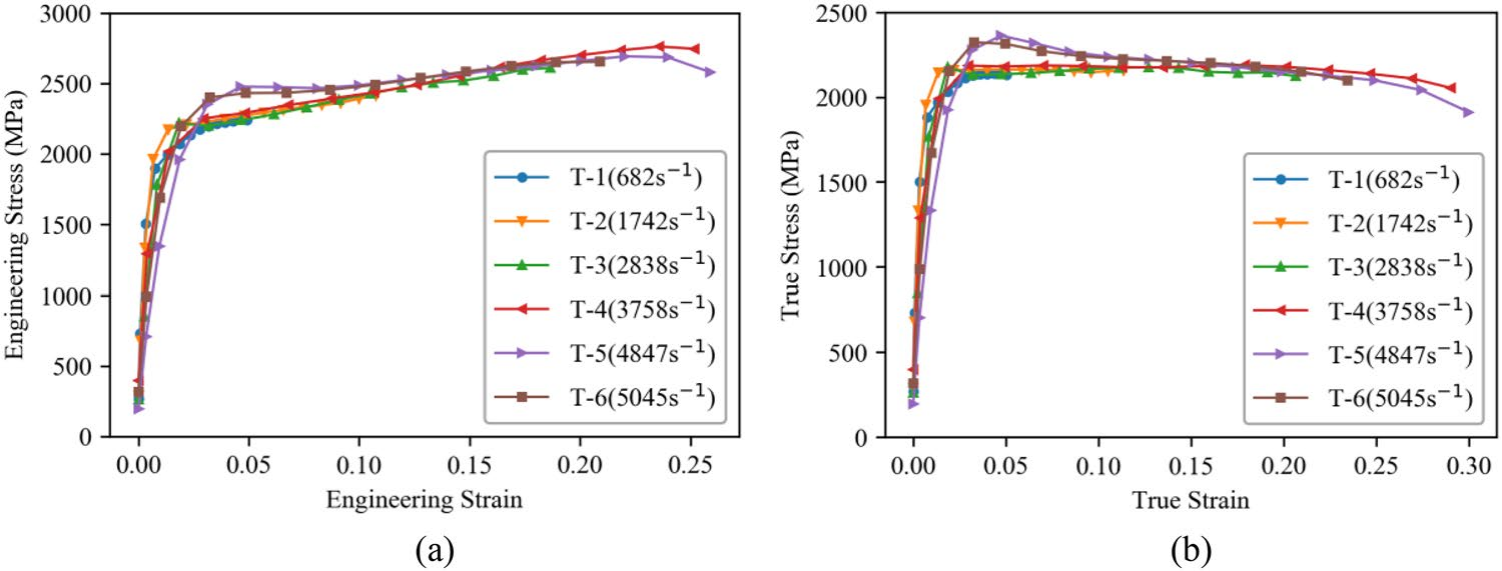
Stress–strain curves of the armor steel from dynamic compression tests: **a** Engineering stress–strain curves, **b** True stress–strain curves.

To determine the dynamic parameter *C*, cylindrical specimens with dimensions of Φ4 × 4 mm were obtained from the same target plate. Subsequently, Split Hopkinson Pressure Bar (SHPB) dynamic compression tests were conducted. Figure 4b presents the initial and deformed specimens from the dynamic compression tests performed at various strain rates ranging from 500 to 5000 s^−1^. As depicted, the magnitude of deformation increases with higher strain rates, and the last two specimens even exhibit cracks. Figure 5 displays the corresponding stress–strain curves obtained from the dynamic compression tests. Similar to the observations in Fig. 3, the true stress is notably lower than the engineering stress, while the true strain is slightly higher than the engineering strain during dynamic compression. Moreover, the mechanical properties of the armor steel are found to be sensitive to the strain rate. Within the range of 500–4000 s^−1^, the yield stress of the armor steel shows a slight increase with higher strain rates. However, beyond a strain rate of 4000 s^−1^, the yield stress suddenly rises to a significantly higher level.

**Figure 6:**
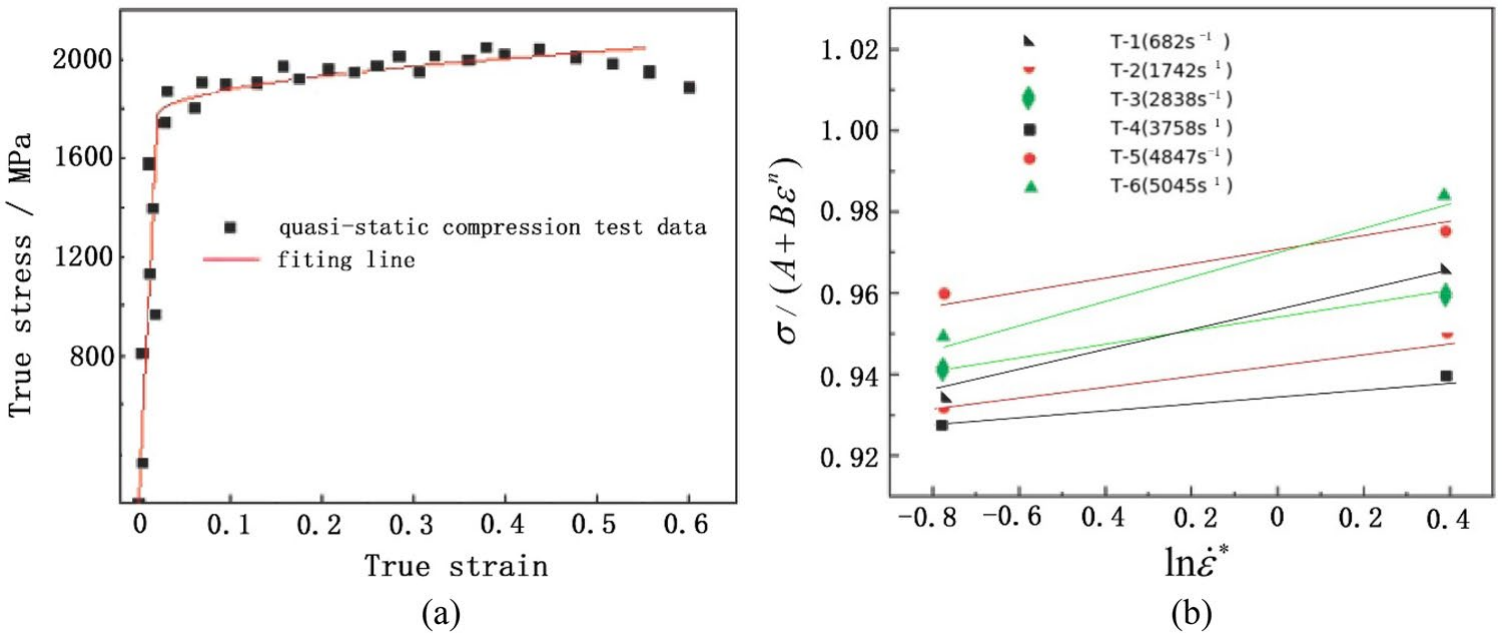
Parameter fitting: **a** Fitting diagram of true stress–strain curve for quasi-compression test, **b** Strain rate strengthening parameter fitting.

Equation (2) can be transformed into Eq. (7) when the plastic strain is zero. By utilizing the quasi-static parameter *A* obtained previously and the calculated results presented in Fig. 5b, the dynamic parameter *C* can be determined by fitting the corresponding data using Eq. (7), as shown in Fig. 6b. The values for parameter C are listed in Table 2.

7 $$ \sigma = A(1 + C{\text{ln}}\dot{\varepsilon }^{ * } ) $$

Considerable research has been conducted by scholars^26,27^ to determine the material constants of the bullet. A comparative analysis reveals that the simulation results obtained using the projectile parameters reported in Kilic’s study^26^ align well with the shooting results. Consequently, the parameters reported by Kilic^26^ are selected for this study, and their values are listed in Table 2.

## Results

In this study, non-linear numerical simulations were performed using LS-DYNA. Hexahedral solid elements were employed to model both the target plate and projectile, as they have been shown to offer superior quality compared to tetrahedral elements^28^. The geometries of the target plate (4.6 mm thick) and the AP bullet closely resemble those used in the shooting tests, with certain details simplified to reduce computation time without compromising accuracy. Since the boundary conditions have minimal influence on penetration resistance during high-speed impact on the target plate, the boundaries were set as fully clamped.

**Figure 7:**
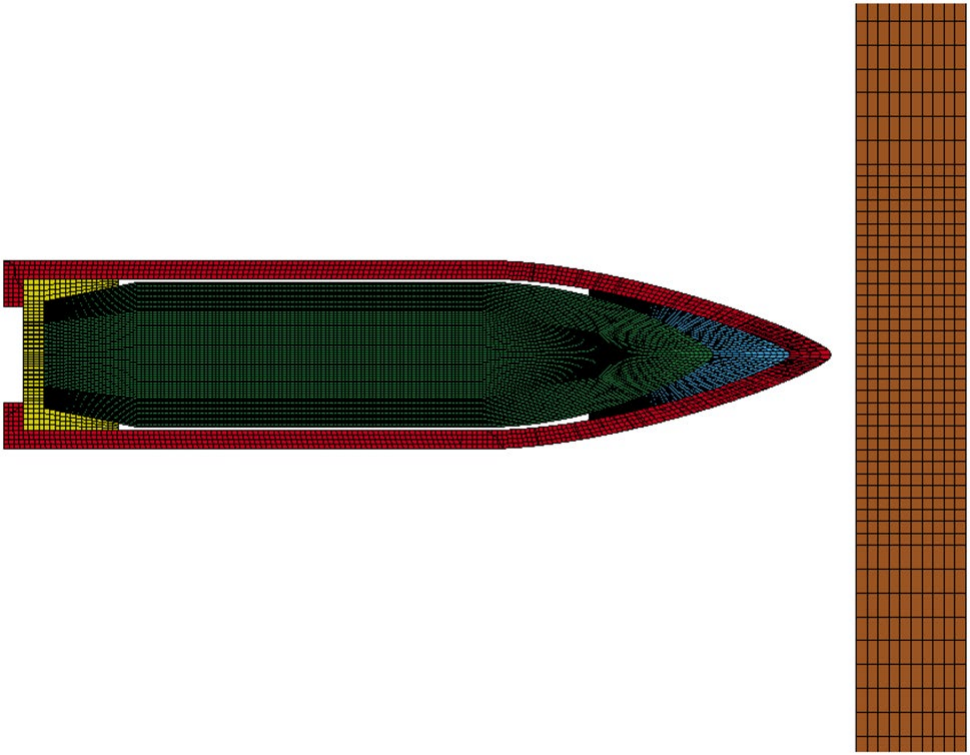
FE model.

Regarding meshing, previous research^29^ suggests that the limit ballistic velocity of the target plate converges when the element size is reduced from 0.5 mm to 0.25 mm. This indicates that further refinement of the mesh does not significantly improve computational accuracy. Based on this finding, a small element size of 0.2 mm was used for the bullet and the inner area of the target plate where deformation is significant, ensuring simulation accuracy. Conversely, a larger mesh element size was employed for other areas of the target plate to reduce computation time. The resulting ballistic finite element (FE) model is depicted in Fig. 7.

**Figure 8:**
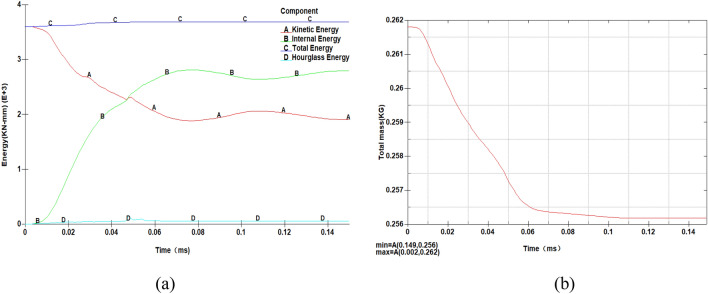
Energy and mass curves to verify the model: **a** Energy curve, **b** Mass curve.

The target plate was subjected to a simulated impact velocity of 820 m/s, and the resulting ballistic responses were analyzed. Figure 8a illustrates the energy curves during the impact process. It can be observed that a small amount of hourglass energy, with a maximum value of 100 kN-mm, emerged during the impact. This accounts for approximately 2.7% of the total system energy, which is well below the requirement of less than 5%^30^. Hourglass mode refers to a zero-energy deformation mode that does not produce strain or stress. It occurs in under-integrated (single integration point) solid, shell, and thick shell elements^31^. Zero-energy modes of deformation do not consume energy and are nonphysical deformation modes. If not controlled, these modes can lead to simulation model instability and meaningless results. Resisting this deformation mode requires a certain amount of energy, known as hourglass energy. A high hourglass energy ratio indicates a significant difference between the calculated deformation model and the actual deformation model, leading to incorrect results. Therefore, it is crucial to minimize and avoid the occurrence of hourglass deformation. An hourglass energy ratio exceeding 10% renders the analysis invalid. Additionally, the kinetic energy curve shows that energy is dissipated as deformation occurs during the impact, resulting in a decrease in kinetic energy, which is rapidly transformed into internal energy. After 0.07 ms, both the kinetic energy and internal energy curves reach a stable state.

Furthermore, in this simulation model, solid elements and an erosion contact algorithm were employed, which may involve element deletion during the calculation process. To ensure successful calculations, the masses of certain parts experiencing significant deformation would sometimes change automatically. Consequently, as the calculation progresses, the overall mass of the model may vary, and the final mass change should be limited to no more than 5% to maintain model stability and ensure valid results. The total mass change during this simulation is presented in Fig. 8b. As observed, the mass of the entire model decreases from 0.262 to 0.256 kg throughout the calculation. Therefore, the maximum mass change during this simulation is approximately 2.3%, satisfying the requirement of not exceeding 5%.

**Figure 9:**
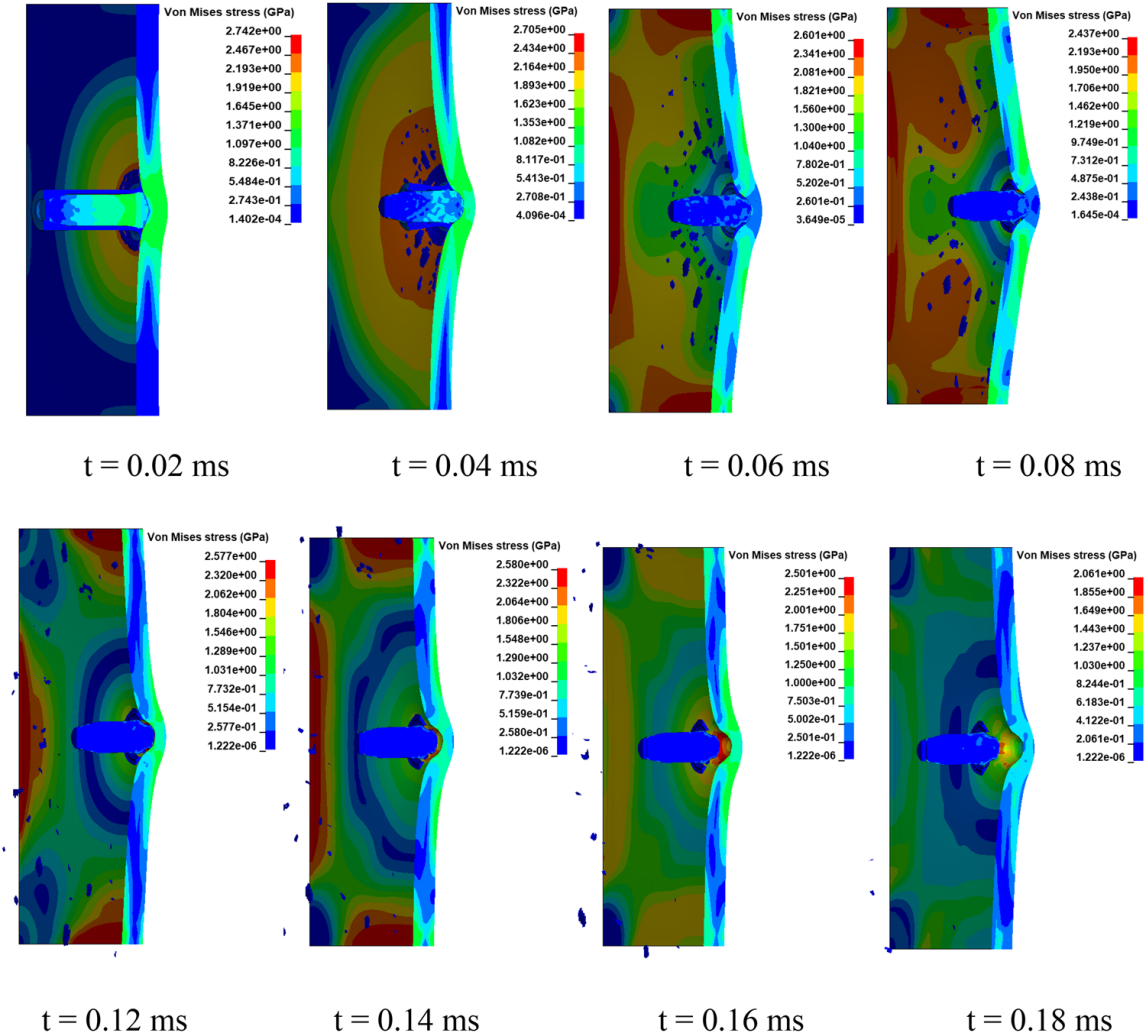
A sequence of Von Mises stress plots at different simulation times (t = 0.02, 0.04, 0.06, 0.08, 0.12, 0.14, and 0.18 ms).

**Figure 10:**
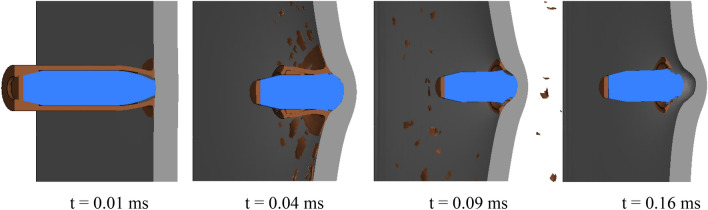
A sequence of plots showing the impacting process at different simulation times (t = 0.01, 0.04, 0.09, and 0.18 ms).

Figure 9 displays the Von Mises stresses at different time instances. The mesh transition between regions is sufficiently accurate to represent stress waves, and no simulation issues, such as mesh distortion, are observed. In the impacted area of the target plate, stress peaks rapidly and then gradually declines in a wave-like manner. Figure 10 illustrates the typical impact process. As depicted, at t = 0.01 ms, erosion occurs at the tip of the bullet’s brass jacket and lead cap due to compression from the hard steel core and the target plate. As the impact continues until 0.04 ms, the brass jacket is stripped from the bullet while the steel core encounters resistance from the target plate, leading to partial destruction of the brass sabot. Concurrently, the continuous compression from the steel core deforms the target plate, resulting in a back bulge. When the impact time reaches 0.09 ms, most parts of the bullet are damaged except for the steel core. At t = 0.16 ms, due to the springback of the target plate, the bullet rebounds and the deformation of the target plate is over. At this point, the crater depth (*H*) can be measured, which amounts to 4.5 mm under the aforementioned impact conditions. Additionally, as shown in the figure, the target plate undergoes deformation but remains unpenetrated, demonstrating its high ballistic resistance against the AP bullet under the impact velocity of 820 m/s.

As mentioned in Sect. 2, the impact velocity employed in this simulation closely matches the ballistic performance test at a shooting distance of 40 m. The average crater depth (*H*) observed in the shooting tests at this distance is 4.17 mm. The relative error between the simulation result (4.5 mm) and the actual shooting test result (4.17 mm) is 7.3%. This significant agreement between the actual shooting result and the simulation result further confirms the validity and accuracy of the established simulation model.

## Discussion

### Single parameter analysis

According to Eq. (1), it is evident that all material constants (*A*, *B*, *n*, and *C*) have an impact on the final equivalent stress of the target plate. Additionally, the equivalent stress significantly affects the ballistic performance. Therefore, in order to assess the extent of influence of these parameters on ballistic performance, it is crucial to clarify the individual impact of each parameter. Using the established simulation model described earlier, the relationships between the J–C parameters of the target plate and its ballistic performance were thoroughly examined through a single parameter analysis. This involved adjusting a specific influencing factor while keeping the other parameters unchanged during ballistic simulation. In this study, the crater depth (*H*) of the impacted target plate was selected as an index to evaluate its corresponding ballistic performance, allowing for a quantitative investigation of the influence of each J–C parameter. As presented in Table 3, for each parameter, four additional values were selected based on the original test values (listed in Table 2) to represent the fluctuation of the mechanical properties of the steel plate, while the other three parameters remained unchanged. Consequently, five results were obtained for each parameter, and a total of 20 simulations were conducted to investigate the influences of these four parameters.

**Table 3:** Single parameter analysis results.

No	*A* (MPa)	*B* (MPa)	*n*	*C*	*H* (mm)
1–1	1500	1050	0.32	0.024	5.27
1–2	1560				4.57
1–3	1630				4.5
1–4	1700				4.17
1–5	1780				3.78
2–1	1630	750	0.32	0.024	5.27
2–2		900			4.92
2–3		1050			4.5
2–4		1200			4.2
2–5		1300			4.03
3–1	1630	1050	0.16	0.024	3.38
3–2			0.24		4.07
3–3			0.32		4.5
3–4			0.4		5.01
3–5			0.48		5.28
4–1	1630	1050	0.32	0.012	5.7
4–2				0.018	5.01
4–3				0.024	4.51
4–4				0.03	4.1
4–5				0.036	3.71

**Figure 11:**
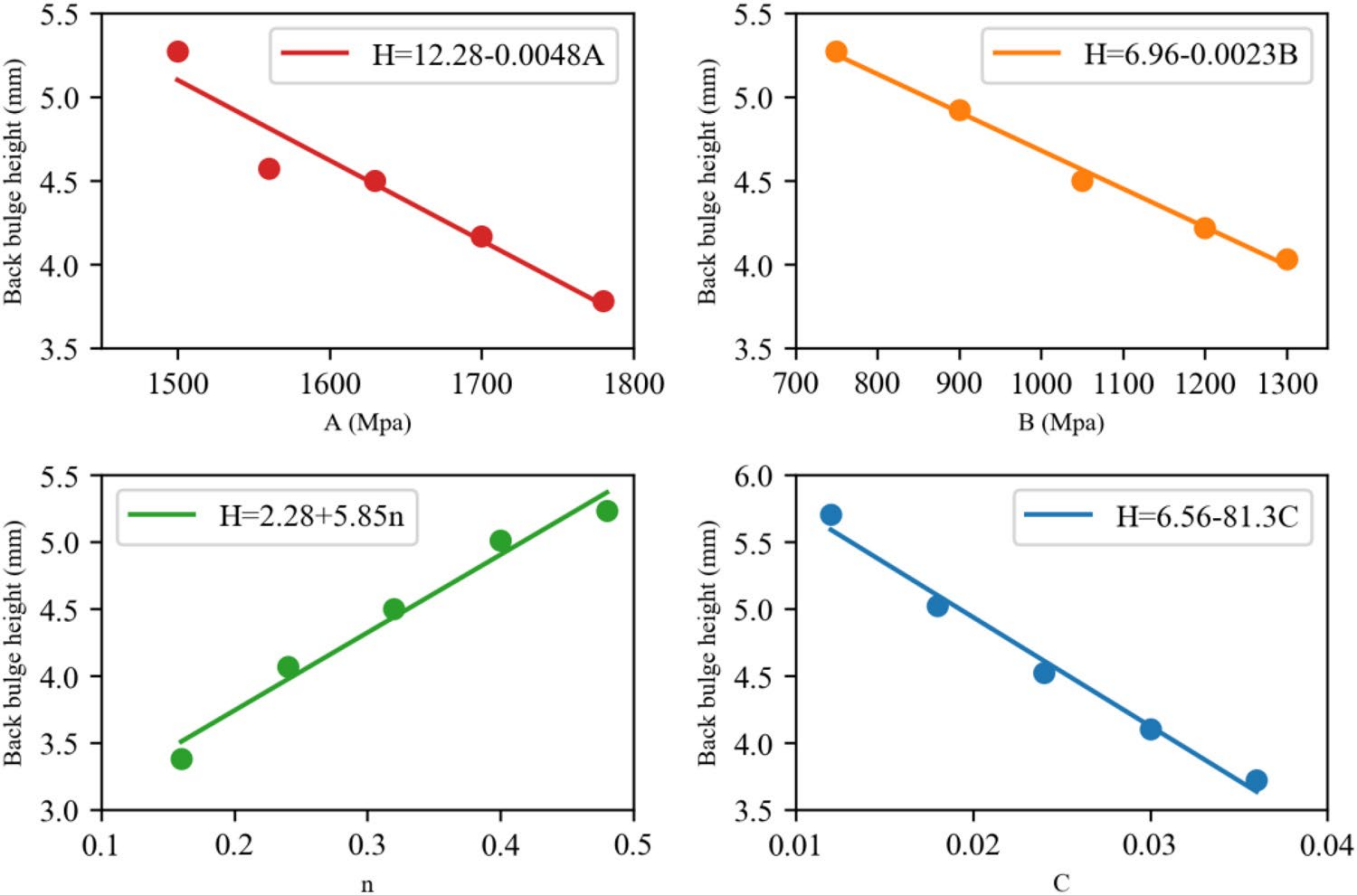
Plots of the relationships between J–C parameters and crater depth.

To provide a clearer illustration of the influences of J–C parameters on ballistic performance, the simulation results presented in Table 3 are plotted in Fig. 11. The corresponding fitting curves and equations, derived using the least square method, are also included. It is evident that linear equations can accurately fit the simulation results and express the relationships between *H* and each J–C parameter within their respective variation ranges.

As depicted in the figure, *H* decreases with an increase in parameter *A*. For the studied armor steels, with similar Young’s moduli, a higher yield stress allows for the absorption of larger impact energy under smaller elastic strains. Furthermore, the increase in strength is a significant factor affecting energy consumption during plastic hole growth. Previous studies^32,33^ have reported that plastic hole growth under vertical high-speed impact can be divided into two stages. The first stage involves penetration and hole growth, where plastic flow occurs on the impacted side as the plastic zone extends to the back. The second stage entails back bulge generation. In both stages, the radial hole expansion force is the main factor influencing plastic hole growth, and the strength of the target plate is directly proportional to the radial hole expansion force. Consequently, enhancing the strength of the target plate increases the energy consumption during plastic hole growth, ultimately reducing the crater depth.

In terms of the strain-hardening parameters *B* and *n*, they exhibit opposite effects on ballistic performance. As observed in the figure, *H* decreases with an increase in *B*, while it increases with an increase in *n*. The negative impact of *n* on ballistic performance can be explained by Eq. (2), where the equivalent plastic strain ***ɛ*** is always less than one. Improved strain hardening capacity increases the normalized hardness value as the target plate deforms, making it more capable of resisting bullet penetration or even fracturing certain parts of the bullet. Studies^34,35^ on adiabatic shear band formation (ASB) have indicated that materials with higher strain hardening rates exhibit lower propensities for ASB formation. Additionally, according to Singh’s study^36^, a higher strain hardening coefficient leads to a larger volume of material involved in absorbing bullet energy through plastic deformation. In other words, a higher strain hardening capacity promotes a wider range of plastic deformation before instability occurs.

Regarding parameter *C*, as depicted in the figure, a higher value of *C* corresponds to a lower crater depth. The strain rate sensitivity significantly influences the ductility and fracture resistance of a material^37–39^. This improvement is often attributed to grain size refinement^40^, grain boundary diffusion (GBD), and grain boundary shear (GBS)^41^. The greater the strain rate sensitivity, the better the elongation and plasticity of the material. Based on this, the strain rate sensitivity parameter *C* plays a positive role in enhancing the ballistic performance of the target plate.

In conclusion, robust linear relationships exist between the crater depth and the four J–C parameters. Hence, linear regression analysis can be employed to quantitatively establish the relationships between them, such as developing a mathematical model for predicting ballistic performance.

### Predictive mathematical model

Although simulation is an effective and rational approach for analyzing target penetration, it often requires a significant amount of computation time to ensure accuracy. Hence, it is crucial to develop an accurate predictive mathematical model that reflects the relationships between ballistic performance and J–C parameters. Multiple linear regression (MLR) models can be obtained by iteratively adjusting regressions with an increasing number of features and selecting the best-fitting model^42^. In this study, based on the observed linear relationships illustrated in Fig. 11, MLR was employed to construct the predictive model.

In this model, the crater depth (*H*) of the target plate serves as the dependent variable, while the independent variables comprise the four aforementioned J–C parameters. As each independent variable (J–C parameter) exerts a linear influence on the dependent variable (*H*), the general regression model can be expressed by Eq. (8).

8 $$ H = \beta_{0} + \beta_{1} A + \beta_{2} B + \beta_{3} n + \beta_{4} C + \varepsilon $$

The regression parameters in Eq. (8) are denoted by *β*_*0*_, *β*_*1*_, *β*_*2*_, *β*_*3*_, and *β*_*4*_, while *ɛ* represents the random error. By manipulating Eq. (8), we can derive Eq. (9).

9 $$ E\left( {H|A,B,n,C} \right) = \beta_{0} + \beta_{1} A + \beta_{2} B + \beta_{3} n + \beta_{4} C $$

In Eq. (9), *E*(*H*|*A*,*B*,*n*,*C***)** represents the estimated value of *H* based on the given independent variables. However, the values of *β*_*0*_, *β*_*1*_, *β*_*2*_, *β*_*3*_, and *β*_*4*_ are typically unknown and need to be calculated using the simulation results. Hence, the sample regression function can be expressed as follows.

10 $$ \mathop H\limits^{ \wedge } = \mathop \beta \limits^{ \wedge }_{0} + \mathop \beta \limits^{ \wedge }_{1} A + \mathop \beta \limits^{ \wedge }_{2} B + \mathop \beta \limits^{ \wedge }_{3} n + \mathop \beta \limits^{ \wedge }_{4} C $$

where $\hat{H}$ is the estimated value of *E*(*H* | *A*,*B*,*n*,*C***).**

Using the simulation results provided in Table 3, a mathematical model expressed as Eq. (11) was developed to predict the crater depth (*H)* under varying J–C parameters. Upon comparing Eq. (11) with the equations presented in Fig. 11, it is evident that the coefficients in Eq. (11) closely align with those in the individual equations. This consistency suggests that the multiple linear regression model accurately captures the collective impact of these four J–C parameters on the ballistic performance of the target plate.

11 $$ \hat{H} = 14.82{ - }0.0048A - 0.0023B + 5.95n - 81.3C $$

12 $$ e_{i} = \mathop H\limits^{{}}_{i} - \hat{H}_{i} $$

13 $$ SD = \sqrt {\frac{1}{N}\sum\limits_{i = 1}^{N} {\left( {e_{i} - \overline{e}} \right)}^{2} } $$

**Figure 12:**
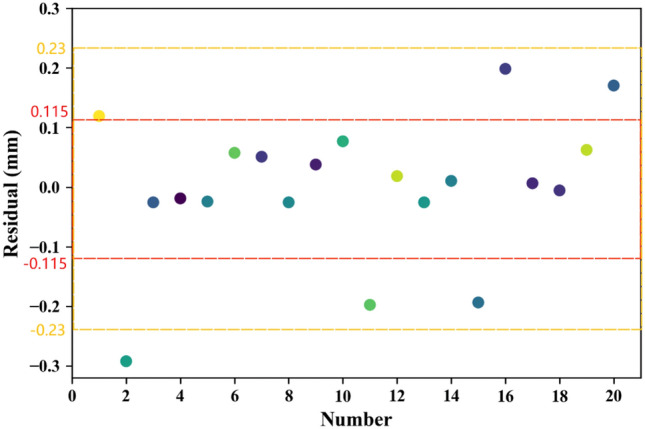
Scatter plot of the estimated residuals by predictive model.

By utilizing the mathematical model and the J–C parameters provided in Table 3, the estimated values of *H* were calculated for each condition. A comparison between the estimated values and the simulated values listed in Table 3 allowed for the determination of residuals (*e*_*i*_) using Eq. (12). The results are presented in Table 4 and depicted in Fig. 12. Moreover, the standard deviation (*SD*), calculated using Eq. (13), is 0.115. Notably, 70% of the residuals fall within the range of − 0.115 mm to 0.115 mm, 95% fall within the range of − 0.23 mm to 0.23 mm, and the largest residual is − 0.292 mm. These findings align closely with the characteristics of a normal distribution, where 68% of variables fall within the range of [-*SD*, *SD*] and 95% fall within the range of [-2*SD*, 2*SD*]. Consequently, this analysis affirms the reliability of the multiple linear regression approach and the validity of the established mathematical model.

**Table 4:** Residuals of the estimated results.

No	1–1	1–2	1–3	1–4	1–5	2–1	2–2	2–3	2–4	2–5
Residuals (mm)	0.119	− 0.292	− 0.026	− 0.019	− 0.024	0.057	0.051	− 0.026	0.039	0.077

No	3–1	3–2	3–3	3–4	3–5	4–1	4–2	4–3	4–4	4–5
Residuals (mm)	− 0.197	0.018	− 0.026	0.010	− 0.194	0.198	0.006	− 0.006	0.062	0.170

To further validate the effectiveness and accuracy of the predictive model, four additional sets of J–C parameters within the initial fluctuation ranges were selected. Utilizing these chosen parameters, the corresponding crater depths were simulated and estimated using both the FE model and the mathematical model. The results obtained from both approaches are presented in Table 5.

**Table 5:** Comparison of simulated and estimated results.

No	*A* (MPa)	*B* (MPa)	*n*	*C*	Simulated *H* (mm)	Estimated* H* (mm)	Relative error (%)
1	1580	900	0.28	0.018	5.45	5.36	1.7
2	1650	1000	0.3	0.02	4.91	4.75	3.3
3	1710	1200	0.4	0.025	4.38	4.19	4.3
4	1760	800	0.45	0.03	4.34	4.75	9.4

Upon comparing the simulated results with those calculated from the mathematical model, it is evident that all relative errors are below 10%. This indicates that the predictive accuracy of the mathematical model exceeds 90%. Thus, the established model effectively captures the relationships between the J–C parameters of the 4.6 mm armor steel and its ballistic performance. Utilizing this mathematical model, the ballistic performance of the armor steel can be predicted in advance, even under conditions of fluctuating mechanical properties during production. Consequently, this model contributes to reducing the cost of shooting tests and saving computation time. Importantly, the methodology employed to establish the predictive mathematical model in this study may also serve as a guiding framework for researching other protective materials.

As observed in Tables 3 and 5, the range of mechanical properties investigated in this study is extensive, encompassing not only the variations that occur in the production of this specific armor steel but also the mechanical properties of other steels. Within this range, the predictive accuracy of the established mathematical model exceeds 90%. However, it should be noted that the relationships between J–C parameters and *H* are only approximately linear, rather than strictly linear as shown in Fig. 11. Consequently, the predictive accuracy may decrease when J–C parameters fall outside the discussed range. It is important to recognize that the ballistic failure type may change as the target material varies. In such cases, the predictive mathematical model should be reevaluated using the aforementioned method. While the re-derived model by MLR will bear some similarity to the original predictive model, the coefficients associated with the J–C parameters may undergo certain changes. Furthermore, since the model has not yet been extensively validated in actual production settings, it necessitates verification and further refinement using a large volume of production data in future studies.

## Conclusions

In this research, the ballistic performance of armor steel was thoroughly investigated, specifically focusing on the effects of J–C parameters. Based on the comprehensive analysis of experimental and simulation results, the following conclusions can be drawn:

1. The J–C constitutive relation for the armor steel under study can be expressed as *σ* = (1630 + 1050*ɛ*^0.32^)(1 + 0.024 ln $\dot{\varepsilon }^{ * }$). It was found that the target plate exhibits exceptional ballistic resistance against AP bullets at an impact velocity of 820 m/s.
2. The ballistic FE-model developed in this study proved to be effective and accurate, demonstrating a strong agreement between the simulated results and those obtained from shooting tests. The relative error in crater depth between the two sets of results was only 7.3%.
3. The investigation of J–C parameters revealed that increasing values of *A*, *B*, and *C* contribute to enhancing ballistic performance, as reflected by a reduction in crater depth (*H*). However, the parameter *n* exhibits an opposite effect. Notably, these relationships exhibit approximate linearity.
4. By employing multiple linear regression analysis, a predictive mathematical model was established, represented by the equation *H* = 14.82 − 0.0048*A* − 0.0023*B* + 5.95*n* − 81.3*C*. The model demonstrated a prediction accuracy exceeding 90%.

## Data Availability

All data generated or analysed during this study are included in this published article.
