# Author Correction: Ballistic analysis of high-performance armor steel by numerical simulation

**DOI:** 10.1038/s41598-024-66733-3

**Published:** 2024-07-08

**Authors:** Defa Li, Feng Huang, Binzhi Ren, Wei Zhang, Junjie Xiong, Binjun Zhou, Xun Guo

**Affiliations:** 1 School of Mechanical and Vehicle Engineering, West Anhui University, Luan, 237012 China; 2 Hubei Key Laboratory of Advanced Technology for Automotive Components, Wuhan University of Technology, Wuhan, 430070 China; 3 School of Metallurgy and Materials Engineering, Chongqing University of Science and Technology, Chongqing, 401331 China

Correction to: *Scientific Reports* 10.1038/s41598-024-62482-5, published online 20 May 2024

The original version of this Article contained an error in the spelling of the author Defa Li which was incorrectly given as Deda Li.

The original Article has been corrected.

